# Grazing exclusion reduced soil respiration but increased its temperature sensitivity in a Meadow Grassland on the Tibetan Plateau

**DOI:** 10.1002/ece3.1867

**Published:** 2016-01-11

**Authors:** Ji Chen, Xuhui Zhou, Junfeng Wang, Tracy Hruska, Weiyu Shi, Junji Cao, Baocheng Zhang, Gexi Xu, Yizhao Chen, Yiqi Luo

**Affiliations:** ^1^ State Key Laboratory of Loess and Quaternary Geology (SKLLQG), and Key Laboratory of Aerosol Chemistry and PhysicsInstitute of Earth EnvironmentChinese Academy of SciencesXi'an710061China; ^2^ University of Chinese Academy of SciencesBeijing100049China; ^3^ Center for Global Change and Ecological ForecastingSchool of Ecological and Environmental SciencesEast China Normal UniversityShanghai200062China; ^4^ State Key Laboratory of Frozen Soil Engineering, Cold and Arid Regions Environmental and Engineering Research InstituteChinese Academy of SciencesLanzhou730000China; ^5^ Department of Environmental Science, Policy, and ManagementUniversity of California at BerkeleyBerkeleyCalifornia94720USA; ^6^ Institute of Global Environmental ChangeXi'an Jiaotong UniversityXi'an710049China; ^7^ Research Institute of Forest Ecology, Environment and ProtectionChinese Academy of ForestryBeijing100091China; ^8^ School of Life ScienceNanjing UniversityNanjing210093China; ^9^ Department of Microbiology and Plant BiologyUniversity of OklahomaNormanOklahoma73019USA; ^10^ Center for Earth System ScienceTsinghua UniversityBeijing100084China

**Keywords:** Grazing exclusion, microbial biomass carbon, plant productivity, soil respiration, temperature sensitivity, Tibetan Plateau

## Abstract

Understanding anthropogenic influences on soil respiration (*R*_s_) is critical for accurate predictions of soil carbon fluxes, but it is not known how *R*_s_ responds to grazing exclusion (GE). Here, we conducted a manipulative experiment in a meadow grassland on the Tibetan Plateau to investigate the effects of GE on *R*_s_. The exclusion of livestock significantly increased soil moisture and above‐ground biomass, but it decreased soil temperature, microbial biomass carbon (MBC), and *R*_s_. Regression analysis indicated that the effects of GE on *R*_s_ were mainly due to changes in soil temperature, soil moisture, and MBC. Compared with the grazed blocks, GE significantly decreased soil carbon release by 23.6% over the growing season and 21.4% annually, but it increased the temperature sensitivity (Q_10_) of *R*_s_ by 6.5% and 14.2% for the growing season and annually respectively. Therefore, GE may reduce the release of soil carbon from the Tibetan Plateau, but under future climate warming scenarios, the increases in Q_10_ induced by GE could lead to increased carbon emissions.

## Introduction

Grassland management is a global concern because it not only affects local herders who depend on productive grasslands for their livelihoods but also the global population through significant feedbacks to climate change (Mcsherry and Ritchie 2013). Many grasslands around the world are currently experiencing unprecedented degradation as a result of enhanced human activities and poor grassland management practices (Milchunas and Lauenroth 1993; Su et al. 2005). Although the causes of grassland degradation are varied and not always known, overgrazing by livestock is commonly thought to be one of the most significant contributing factors (Li et al. 2013c). Degradation is ittcqga a vague term (Harris 2010) which includes several possible problems, including reductions in forage production, expansion of sandy areas, and soil erosion (Liu et al. 2008; Wen et al. 2013). Currently, grazing exclusion (GE) is one restoration method that has been broadly applied, especially in China (Jing et al. 2013; Lu et al. 2013; Mcsherry and Ritchie 2013; Laurenson and Houlbrooke 2014; Chen et al. 2015). Despite its wide application, however, the effects and efficacy of this remediation technique on ecosystem functions remain unclear.

Soil respiration (*R*_s_) is the largest flux of carbon exchange between the soil and the atmosphere, and even small fluctuations in *R*_s_ can result in significant variations in the soil carbon budget (Luo and Zhou 2006). There are also evidences suggesting that small increases in terrestrial *R*_s_ can result in significant higher atmospheric CO_2_ concentration, and, ultimately, feedbacks to global climate change (Luo and Zhou 2006; Vargas et al. 2011). It also has been shown that the amount of carbon stored in grassland soils is influenced by grassland management practices, especially grazing (Conant et al. 2001; Maia et al. 2009; Mcsherry and Ritchie 2013; Cao et al. 2015). Therefore, understanding how GE affects *R*_s_ would significantly contribute to our knowledge of grassland soil carbon flux and inform grassland management techniques to combat climate change.

The underlying mechanisms affecting the responses of *R*_s_ to GE are still not fully understood, but is well‐documented that *R*_s_ increases exponentially with soil temperature (Laganière et al. 2012; Suseela et al. 2012). Furthermore, GE has been found to significantly decrease soil temperature compared to grazed plots due to increased canopy coverage (Susiluoto et al. 2008; Schoenbach et al. 2012). In addition, GE can modify *R*_s_ by altering plant biomass accumulation; for example, one field experiment along a desert steppe in northern China showed that GE increased *R*_s_ in proportion to the increases in above‐ground biomass (AGB) and below‐ground biomass (BGB) (Hou et al. 2014). Soil microbial activity also can be affected by GE through its effects on soil temperature and plant productivity (Lu et al. 2013; Nianpeng et al. 2013; Zou et al. 2013). Indeed, a related field experiment conducted on the Tibetan Plateau found that GE profoundly repressed abundances of genes involved in carbon‐degradation due to a corresponding increases in AGB and soil substrate availability (Yang et al. 2013). *R*_s_, especially heterotrophic respiration, is strongly associated with microbial communities and their activities (Suseela et al. 2012). Although these studies provide evidence that GE may have profound effects on *R*_s_, our understanding of the responses of *R*_s_ to GE is still hampered by our poor knowledge regarding the driving mechanisms.

The temperature sensitivity of *R*_s_ as indicated by Q_10_ − as used here a measure of the rate of change in *R*_s_ as a consequence of raising the temperature by 10°C−links the response of *R*_s_ to global climate change (Zhou et al. 2007, 2009). Many current land‐surface carbon‐climate models are based on this parameter (Davidson et al. 2012; Suseela et al. 2012; Hamdi et al. 2013), but few studies have investigated the response of Q_10_ to GE, and the results to date have been inconclusive. One incubation experiment showed that GE decreased Q_10_ (Paz‐Ferreiro et al. 2012) while another field study indicated that GE increased Q_10_ (Lin et al. 2011). Although the effects of GE on Q_10_ are still unclear, the conflicting results suggest that there may be interactive effects of climate warming and GE on *R*_s_, and therefore, the effects of GE on Q_10_ should be incorporated into models of the effects of human activities on *R*_s_. Indeed, understanding how Q_10_ responds to GE is a key to predicting the feedbacks between *R*_s_ to GE, but this is currently limited by the paucity of relevant studies.

As one of the highest and youngest Plateau in the world, the Tibetan Plateau extends over 2.5 million km^2^ (Zhang and Welker 1996). The majority of the Plateau stands ≥ 3000 m above sea level, and this high elevation makes it particularly susceptible to disturbances caused by human activities. Covering an area of 1.2 × 10^6^ km^2^ and accounting for about 48% of the Plateau's land area (Hu et al. 2004), the alpine meadow grassland is one of the most widespread types of vegetation on the Plateau. The CO_2_ exchange between the meadow grassland and the atmosphere has been the focus of carbon‐flux research due to its critical effects on the East Asian summer monsoon (Risch and Frank 2010). Moreover, the Tibetan Plateau is currently experiencing an intensification of human activities, including grazing, and a relatively high temperature increase (IPCC, 2014), rendering the great quantities of soil carbon susceptible to release (Ward et al. 2014). The Chinese government is currently imposing GE in areas over one‐third of the Tibetan Plateau (Cencetti 2011; Yu and Farrell 2013), but it is largely unknown how GE will affect the release of soil carbon.

To address these knowledge gaps, we conducted a field manipulation experiment in a meadow grassland on the Tibetan Plateau to investigate the effects of GE on *R*_s_. Our aims were to (1) to determine how soil temperature, soil moisture, microbial biomass carbon (MBC), AGB, BGB and *R*_s_ respond to GE; (2) to explore the underlying mechanisms associated with the responses of *R*_s_ to GE; and (3) to evaluate the effects of GE on Q_10_.

## Materials and Methods

### Study site

**Figure 1:**
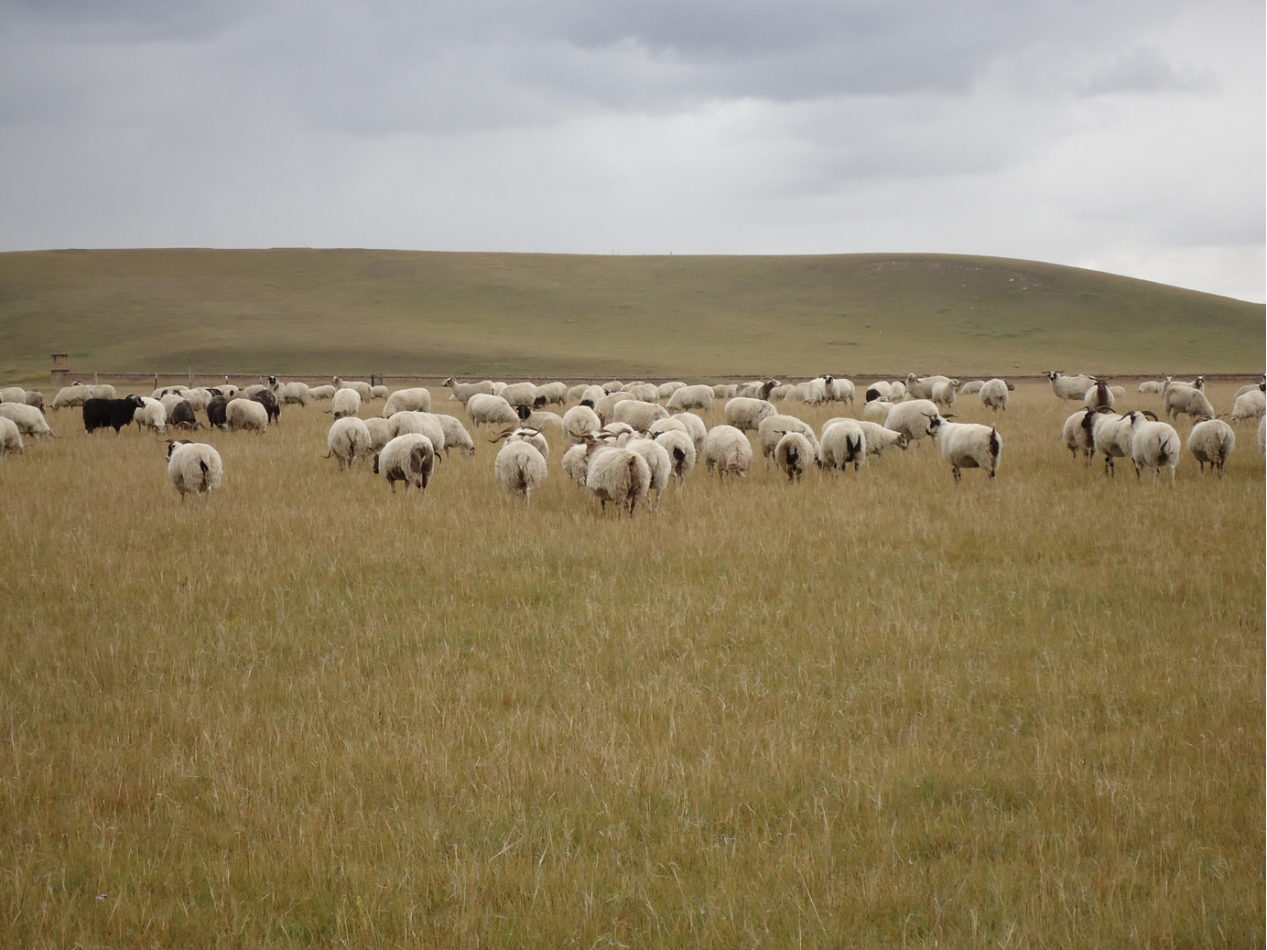
The studied grassland on the Tibetan Plateau.

This study was conducted at the Haibei Grassland Ecological Monitoring Station of the China Meteorological Administration, which is in the town of Xihai, Haiyan County, Haibei Prefecture, Qinghai, China (100°51′E, 36°57′N, 3140 m). The study area has a typical plateau continental climate. The average annual precipitation from 1976 to 2010 was 398.2 mm, with 85% of the rainfall falling during the growing season from May to October. The average annual air temperature is 0.8°C, and the monthly mean air temperature ranges from 13.4 in July to −14.2°C in January. The study site has a sandy loam soil texture, and the soils were classified as mountain brown according to the Chinese soil classification system or as cambisols in the Food and Agriculture Organization classification scheme (Klein et al. 2005). The vegetation is typical meadow grassland dominated by *Stipa sareptana var. krylovii, Stipa purpurea, Koeleria cristata*,* Poa crymophila*,* Kobresia humilis*,* Artemisia scoparia*,* Aster tataricus*, and *Medicago ruthenica* (Fig. 1) (Chen et al. 2015).

### Experimental design

Twelve 60 m × 30 m blocks were selected for this study in April 2012. Six blocks were placed in a pasture that had been fenced in October 2007 to exclude grazing, and the other six blocks were in an adjacent free‐grazing pasture. Buffer zones 5–10 m wide were set between adjacent blocks. Before GE, all blocks had been freely grazed as winter pasture which typically occurred from the end of September to early April. For the GE treatment, the livestock was completely excluded from six blocks. The stocking density of the animals in the grazing pasture was 0.5 yak and 2.5 sheep per hectare which is equivalent to 1.41 tropical livestock units ha^−1^.

### Soil respiration measurements

At the commencement of the field measurements, polyvinyl chloride collars (PVC, 20 cm in internal diameter and 5 cm in height) were randomly inserted 2−3 cm into the soil in each block to measure *R*_s_. All above‐ground plant material was removed before the PVC collars were inserted into the soil for both the grazing and GE treatments. After that, all living plants inside the PVC collars were clipped at least 1 day before *R*_s_ measurements in order to exclude plant respiration (Zhou et al. 2007), but the clipped plant material was left in the collars to decompose. During the growing season, variations of *R*_s_ were measured at least twice each month, and diurnal cycles of *R*_s_ were measured at 2‐h intervals from 08:00 to 20:00 (i.e., 08:00, 10:00, 12:00, 14:00, 16:00, 18:00, and 20:00, local time). During the nongrowing season (NGS), measurements were collected once per day between 8:00 and 12:00 local time. Our data has shown that measurements taken between 8:00 and 12:00 are a good proxy for diurnal mean values (Chen et al., 2015). A soil CO_2_ Flux Chamber was attached to a gas analyzer (LI‐8100; LI‐COR, Inc., Lincoln, NE) for 2 min on each collar to measure *R*_s_ and then moved to the next collar. All measurements were carried out on sunny days.

### Plant biomass

Above‐ground biomass was determined by weighing all vegetation clipped from six 0.5 m × 0.5 m quadrats in each block. BGB was measured by collecting six replicate soil samples (4 cm in diameter) from depths of 0−40 cm. Our previous results showed that >95% of the total BGB is distributed in the upper 0−40 cm of the soils (Chen et al. 2015). Both above‐ and below‐ground plant material was washed and then oven dried at 65°C for 72 h before being weighed. AGB and BGB were collected from all 12 blocks on 14 May, 19 June, 9 July, 15 August, 8 September, and 14 October in 2012.

### Soil sampling and microbial biomass carbon

Soil samples were collected at a depth of 0−10 cm at the same time as the BGB samples were collected. For each block, we removed surface litter and collected soil from 0 to 10 cm with the use of a soil auger (4 cm in diameter). Soil samples were cleaned of plant roots and large stones using a 2‐mm sieve, and then all visible plant matter was manually removed from the sieved samples. The samples were then packed into a portable refrigerated box and transported to the laboratory where they were stored at 4°C and analyzed within 2 weeks.

Microbial biomass carbon was measured by the chloroform fumigation‐extraction method (Brookes et al. 1985). Briefly, a 10 g moist soil field sample was fumigated with chloroform for 24 h and extracted with 0.5 mol·L^−1^ K_2_SO_4_ in an end‐to‐end shaker for 1 h, then the supernatant was filtered. Meanwhile, a second 10 g moist soil sample was not fumigated but directly extracted as described above. The amounts of total carbon in the fumigated and un‐fumigated soil extracts were determined using a TOC analyzer (Multi N/C 3100; Analytik, Jena, Germany). To account for incomplete extraction, we used an extraction efficiency factor of 0.45 following (Brookes et al. 1985). The MBC concentrations were calculated as the difference between fumigated and un‐fumigated samples.

### Soil temperature and moisture

Soil temperature and soil moisture were recorded with HOBO data loggers (Onset Computer Company, Pocasset, MA). Soil temperature was measured using a thermocouple probe while the soil moisture content was measured using gypsum cast around two concentric stainless‐steel electrodes (Delmhorst Instrument Co., Towaco, NJ). Data loggers recorded average soil temperature and soil moisture every 5 min. Soil temperature was measured for the whole experimental period, which was from April 2012 to May 2013. Soil moisture data were unavailable during the NGS due to consistent freezing temperatures.

### Data analysis

Monthly variations in *R*_s_ were calculated from the daily arithmetic mean values, which were the averages of all measurements made during the same day. Two‐way analyses of variance (ANOVA) were applied to examine the effects of GE, measuring date, and their interactions on *R*_s_. One‐way ANOVAs were used to compare the differences in AGB, BGB, MBC, and Q_10_ between the grazing and GE blocks. Significant differences were evaluated at a probability level of *P *<* *0.05. Linear or exponential regression analyses was used to assess the influence of MBC, soil temperature, and soil water content on *R*_s_. Multiple linear regression analysis was used to evaluate the overall effects of AGB, BGB, MBC, soil temperature, and soil moisture on *R*_s_. The goodness of fit relative to the number of model parameters was evaluated by the Akaike information criterion (AIC). The model with the smallest AIC value is regarded as the most likely representation of the truth (Akaike 1974; Burnham and Anderson 2004). GE‐induced variations in *R*_s_ refer to a calculation of *R*_s_ in the GE blocks minus the corresponding *R*_s_ value for the grazed blocks.

An exponential function was applied to assess temperature sensitivity of *R*_s_ by: *R* = α*e* ^b*T*^. Where *R* is the *R*_s_, *T* is the soil temperature, α is the intercept of respiration when the temperature is 0°C, and b is a constant that was used to calculate Q_10_ using the following function (Zhou et al. 2007): Q_10_ = *e* ^10b^. Carbon released from soil was calculated by multiplying daily values of *R*_s_ and the number of days between measurements (Niu et al. 2010).

## Results

### Variations in biotic and abiotic factors

**Figure 2:**
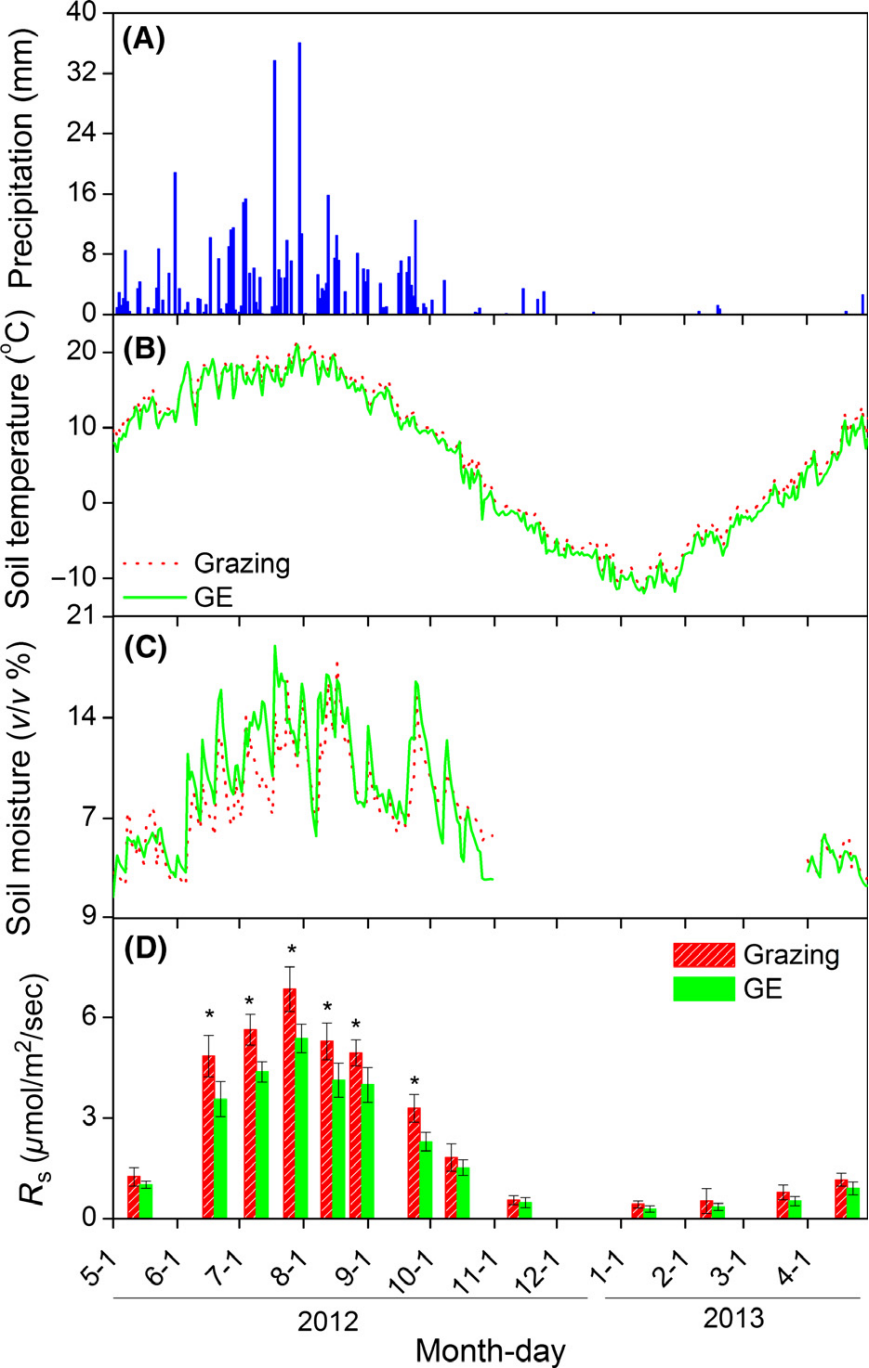
Annual variations of (A) precipitation, (B) soil temperature, (C) soil moisture, and (D) soil respiration (*R*_s_) for the grazing and grazing exclusion (GE) treatments. Vertical bars indicate arithmetic means ± standard errors for six replicates. Asterisks indicate significant differences for GE effects on *R*_s_ at a probability of *P *<* *0.05).

Grazing exclusion significantly increased soil moisture, but GE decreased soil temperature relative to the grazed plots during the growing season (Fig. 2). The highest soil temperature and moisture readings occurred from mid‐June to mid‐August. The mean soil temperature during the growing season was 15.1 and 14.7°C for the grazing and GE blocks, respectively. The mean soil moisture during the growing season was 9.1% and 10.0% for grazing and GE respectively (Fig. 2). During the NGS, no significant difference between blocks was found for soil temperature.

**Figure 3:**
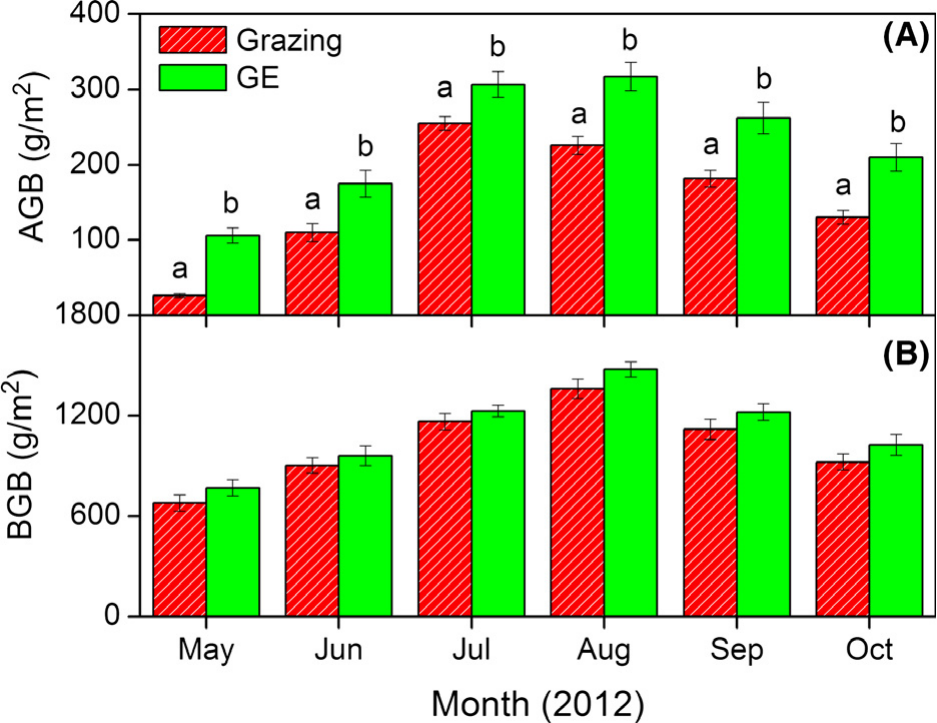
Variations of (A) above‐ground biomass (AGB) and (B) below‐ground biomass (BGB) for the grazing and grazing exclusion (GE) treatments. Vertical bars indicate arithmetic means ± standard errors for six replicates; different lowercase letters indicate a significant difference between grazing and GE at a probability *P *<* *0.05. No significant differences were found for BGB.

**Figure 4:**
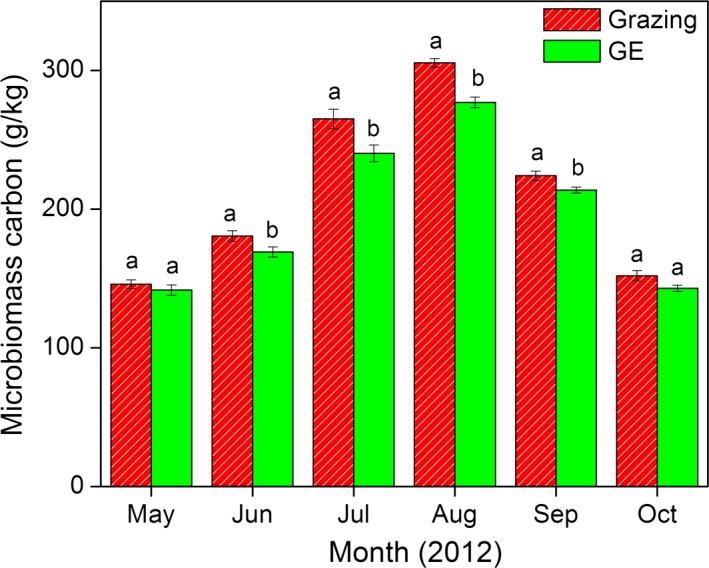
Variations of microbial biomass carbon (MBC) for grazing and grazing exclusion (GE) treatments. Vertical bars indicate arithmetic means ± standard errors for six replicates; different lowercase letters indicate a significant difference between grazing and GE while the same letters indicate no statistical difference (*P *<* *0.05).

Above‐ground biomass increased rapidly from early May to late July when peak values of 251.08 g·m^−2^ were recorded for the grazing block and 316.6 g·m^−2^ for the GE block. Compared with the grazed blocks, GE significantly increased AGB during the growing season. Although BGB also increased from May to August, no significant GE effect was found for BGB (Fig. 3). Compared with grazing, GE significantly reduced MBC by 6.3%, 9.3%, 9.3%, and 4.6% in June, July, August, and September respectively (Fig. 4).

### Variations in soil respiration

**Figure 5:**
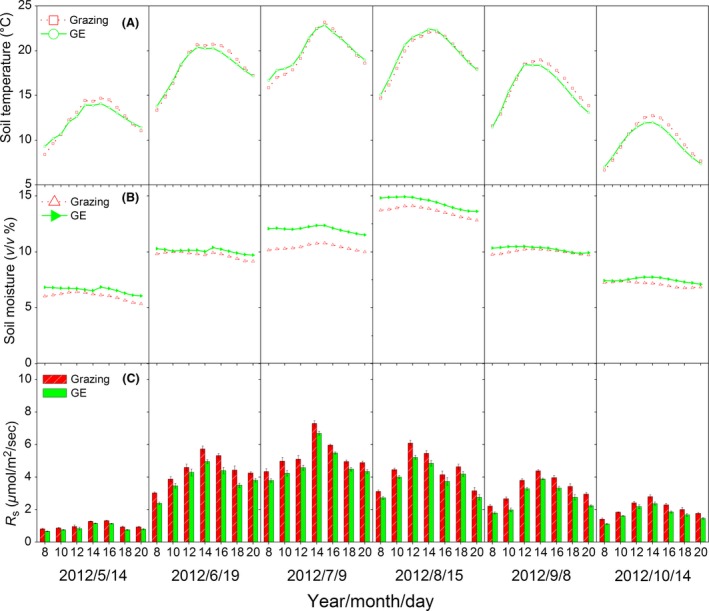
Daily variations of (A) soil temperature, (B) soil moisture at 10 cm depth, and (C) soil respiration (*R*_s_) for grazing and grazing exclusion (GE) treatments during the growing season. Vertical bars show ± standard errors for six replicates. Values in the *x*‐axis indicate the hours.

The daily patterns of *R*_s_ during the growing season were similar between the grazing and GE blocks (Fig. 5), and for both blocks, the daily peak *R*_s_ values coincided with peaks in the daily soil temperatures. Over the longer term, *R*_s_ was highest during July and August (Fig. 2D), coincident with the highest soil temperature and moisture values. Annually, *R*_s_ ranged from 0.42 to 6.05 *μ*mol·m^−2^·sec^−1^ for the grazing block compared with 0.28 to 5.38 *μ*mol·m^−2^·sec^−1^ for the GE block. *R*_s_ varied less during the NGS, ranging from 0.42 to 1.16 and 0.28 to 0.90 *μ*mol·m^−2^·sec^−1^ for grazing and GE blocks, respectively (Fig. 2D). Compared with grazing, GE significantly decreased *R*_s_ during the growing season as well as annually (Table 1). Monthly differences in the mean *R*_s_ values were statistically significant, and there were significant interactive effects between GE and measurement date on *R*_s_ both during the growing season and annually (Table 1).

**Table 1:** Results (*P* values) of two‐way analyses of variance on the effects of grazing exclusion (GE), measuring date (*D*), and their interactions on soil respiration

Effect	Growing season	Non‐growing season	Annual
*D*	***P *** **<** *** *** **0.001**	0.461	***P *** **<** *** *** **0.001**
GE	**0.015**	0.655	**0.034**
*D* × GE	***P *** **<** *** *** **0.001**	0.766	**0.007**

values in bold indicate significant difference.

**Figure 6:**
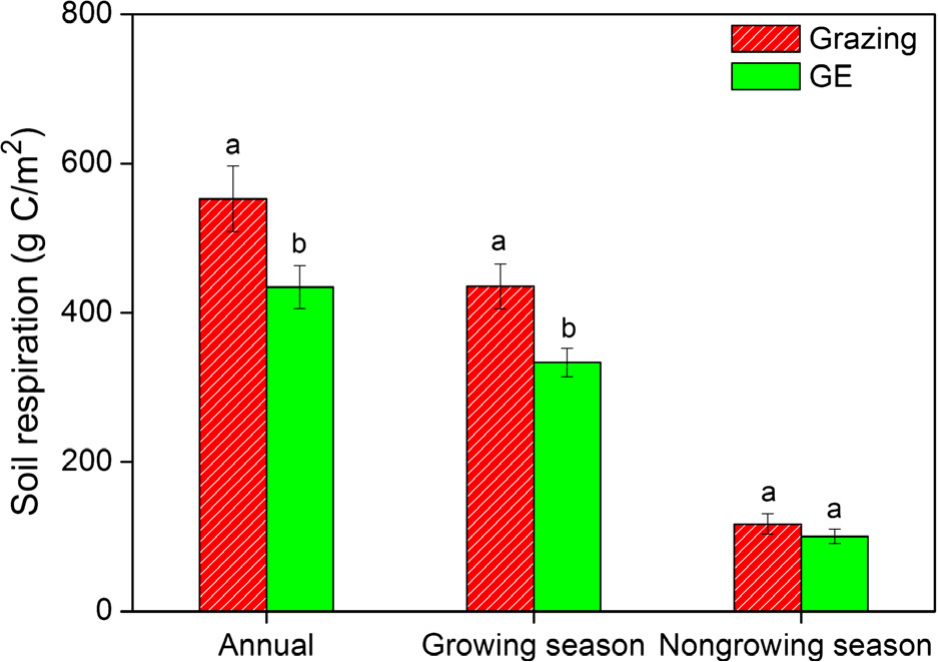
The amount of carbon released by soil respiration (*R*_s_) annually and for the growing season and nongrowing season for the grazing and grazing exclusion (GE) treatments. Vertical bars indicate mean ± standard errors for six replicates; different lowercase letters indicate a significant difference between grazing and GE while the same letters indicate no statistical difference (*P* < 0.05).

Grazing exclusion decreased the release of soil carbon by 21.40% annually and 23.36% during the growing season. Although there was no significant GE effect on *R*_s_ during the NGS, the proportion of carbon released by *R*_s_ during that period was 21.20% and 23.16% of annual totals for the grazing and GE blocks, respectively (Fig. 6).

### Factors affecting soil respiration

**Figure 7:**
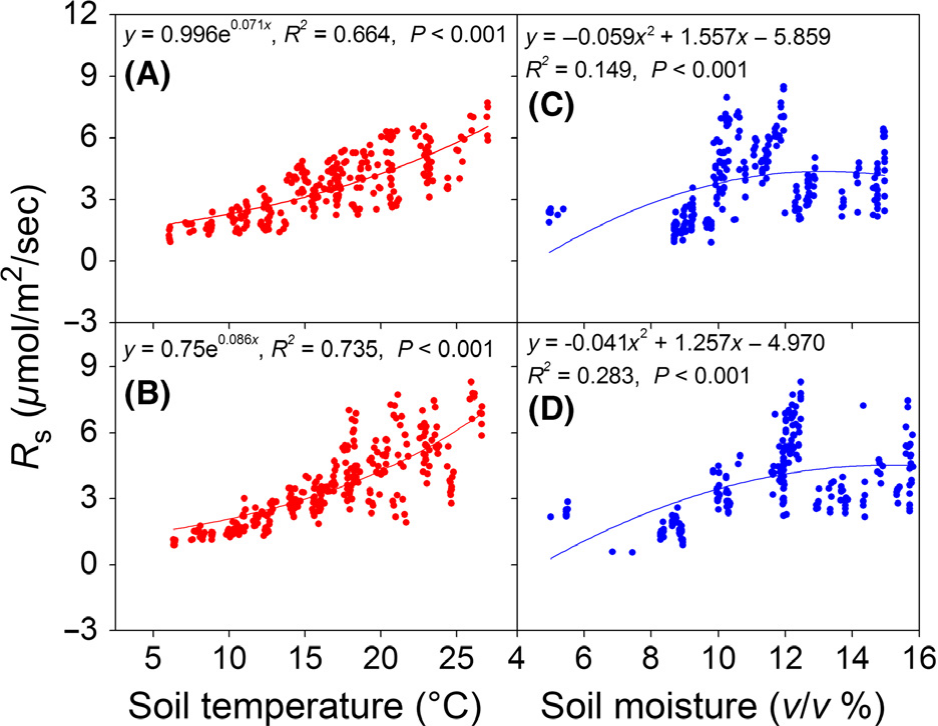
Relationships between soil temperature, soil moisture and soil respiration (*R*_s_) for the grazed blocks (A and C) and the grazing exclusion (GE) blocks (B and D).

**Figure 8:**
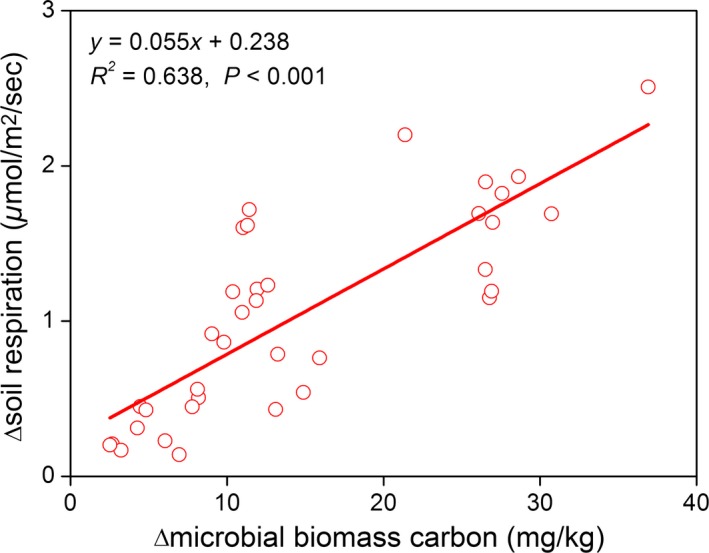
Relationship of grazing exclusion (GE) induced variations in soil respiration (*R*_s_) and microbial biomass carbon (MBC). The data used are only for the growing season because MBC was measured only during that season.

*R*_s_ was significantly correlated with soil temperature and moisture during the growing season (Fig. 7 and Table 2), and regression analysis showed that changes in soil temperature accounted for 66.4% and 73.5% of the seasonal variations of *R*_s_ for grazed and GE blocks respectively. Soil moisture accounted for another 14.9% and 28.3% of the respective variance (Fig. 7). Pearson's correlation analysis showed that GE‐induced reductions in *R*_s_ were mainly associated with co‐occurring changes in MBC and soil temperature (Table 2); indeed, MBC alone accounted for 63.8% of the variations of *R*_s_ during the growing season (Fig. 8). In addition, our regression analysis showed that the best model for the effects of GE‐induced variations on *R*_s_ included soil temperature, soil moisture, and MBC as predictors (Table 3).

**Table 2:** Results (*P* value) of Pearson's correlation analyses of grazing exclusion induced variations in soil temperature (ST), soil moisture (SM), microbial biomass carbon (MBC), above‐ground biomass (AGB), below‐ground biomass (BGB) and soil respiration (*R*_s_). Values in italic indicate negative correlation, values in bold indicate significant correlations at *P *<* *0.05

	ST	SM	MBC	AGB	BGB	*R*_s_
ST	1.000					
SM	*0.704*	1.000				
MBC	0.426	*0.407*	1.000			
AGB	*0.511*	0.306	*0.914*	1.000		
BGB	*0.616*	0.714	*0.508*	0.709	1.000	
*R*_s_	**<0.001**	*0.521*	**0.006**	*0.897*	*0.687*	1.000

**Table 3:** Multiple linear regression analysis of the interactive effects of grazing exclusion‐induced changes in both biotic and abiotic factors on soil respiration (*R*_s_). Plus sign (+) indicates entered variables. Akaike information criterion (AIC). See Table 2 for other abbreviations

Variable	ST	SM	MBC	AGB	BGB	*R* ^*2*^	*P*	AIC
*R*_s_	+	+				0.378	<0.001	−25.800
*R*_s_	+		+			0.508	<0.001	−34.268
*R*_s_		+	+			0.234	n.s.	−18.304
*R*_s_	+	+	+			0.538	<0.001	−34.514
*R*_s_				+	+	0.005	n.s.	−8.904
*R*_s_			+	+	+	0.202	n.s.	−14.841
*R*_s_	+	+	+	+	+	0.542	<0.001	−30.843

### Temperature sensitivity of soil respiration

Q_10_ values were significantly higher for both the GE and grazing blocks during the growing season compared with the NGS (Table 4). During the former, Q_10_ values for all blocks ranged from 1.67 to 4.12, and higher values found at the start and end of the growing season compared with the peak growing season. During the NGS, Q_10_ values from all blocks ranged from 1.62 to 1.84. GE significantly increased Q_10_ values relative to grazing both during the growing season and annually. Significant differences between GE and grazing also were found during the start (May) and end (September and October) of the growing season, but there was no difference during the peak of the growing season (June, July, and August) or during the NGS (Table 4).

**Table 4:** Temperature sensitivity of soil respiration for the grazing and grazing exclusion (GE) blocks

Period	Grazing	GE
Annual	**2.53** ± **0.25**	**2.89 ± 0.21**
NGS	1.62 ± 0.11	1.84 ± 0.21
Growing season	**3.25 ± 0.15**	**3.46 ± 0.13**
May	**3.53 ± 0.08**	**4.12 ± 0.26**
June	2.59 ± 0.34	3.06 ± 0.40
July	1.67 ± 0.14	2.21 ± 0.48
August	2.30 ± 0.40	3.02 ± 0.52
September	**2.36 ± 0.23**	**3.64 ± 0.17**
October	**2.80 ± 0.32**	**3.22 ± 0.30**

Values are arithmetic mean ± standard error for six replicates. Bold values indicate a significant difference between grazing and GE (*P *<* *0.05).

## Discussion

### Grazing exclusion effects on biotic and abiotic characteristics

Grazing exclusion can have significant effects on an ecosystem's biotic and abiotic characteristics, and these in turn can affect soil carbon fluxes (Mcsherry and Ritchie 2013). In our study, GE significantly decreased soil temperature but increased soil moisture during the growing season (Fig. 2), and these findings are consistent with previous studies in similar areas of the Tibetan Plateau (Cao et al. 2004; Klein et al. 2005). Increased canopy coverage (Chen et al. 2015) was likely to be the primary driver for the lower soil temperatures but higher soil moisture associated with GE. This was largely due to the higher canopy coverage resulting in lower soil surface irradiance and evaporation in the GE sites compared with these in grazing sites (Klein et al. 2005).

Grazing exclusion also resulted in significantly higher AGB but not BGB (Fig. 3). Variations of both AGB and BGB were within the ranges reported previously (Yang et al. 2009; Jing et al. 2013). Increased AGB was the most obvious result of excluding the livestock, and this also likely due to increases in the coverage of some perennial grass species (e.g., short *Kobresia* sp.*, K. cristata*, and *Poa angustifolia L*, unpubl. data, J. Chen, X. Zhou, and J. Cao). Other possible explanations for the increased AGB under GE could be related to GE‐induced changes in plant phenology (Han et al. 2015), enhanced soil nutrient cycles (Lu et al. 2015), or altered plant community (Kohyani et al. 2011). The nonsignificant response of BGB to GE is consistent with earlier short‐term GE studies (Medina‐Roldán et al. 2012; Koerner and Collins 2014). One possible explanation was that the short duration of GE in the current study was insufficient to have significant impacts on BGB, since positive responses were usually found in long‐term GE studies (Cheng et al. 2011; Garcia‐Pausas et al. 2011). The lack of significant increases in BGB to GE may also be related to the reduction in livestock excreta inputs and consequently altered nutrient cycles, a hypothesis with some support from other studies on the Tibetan Plateau (Fu et al. 2014) and Inner Mongolia (Liu et al. 2012).

### Effects of grazing exclusion on soil respiration

Grazing exclusion significantly decreased *R*_s_ both during the growing season and annually (Table 1). GE‐induced changes in soil temperature and moisture had significant impacts on *R*_s_ (Fig. 7, and Table 2), and this is consistent with results from other regions where reductions in *R*_s_ following GE were likely driven by changes in these variables (Paz‐Ferreiro et al. 2012; Gong et al. 2014). A possible explanation for these findings is that virtually all biogeochemical processes associated with *R*_s_ are inextricably linked to soil temperature and moisture (Risch et al. 2013). The seasonal variations in *R*_s_ also followed the patterns of precipitation (Fig. 2), and the impacts of precipitation on *R*_s_ have been reported in previous interannual studies (Liu et al. 2009; Lu et al. 2015). Given the strong impacts of precipitation on *R*_s_, future long‐term studies should examine the interactive effects of precipitation and grazing on *R*_s_.

Reductions in MBC are another factor that likely contributed to the lower *R*_s_ following GE (Fig. 8), and this was independent of the changes in soil temperature and moisture (Table 2). Previous studies have reported that *R*_s_ and MBC were closely related (Suseela et al. 2012; Peng et al. 2014), and this can be explained by the fact that soil heterotrophic respiration, which accounts for more than half of total *R*_s_ in most ecosystems, is mainly the result of microbial growth and decomposition (Tucker et al. 2013; Peng et al. 2014). Livestock excreta inputs might be a contributing factor to higher MBC in grazed blocks due to their critical roles in supplying labile substrates to fuel microbial growth and metabolic activities (Liu et al. 2012; Wang et al. 2013). Other studies also have found that MBC can be reduced as a result of depleted labile soil carbon (Song et al. 2012; Tucker et al. 2013). Our results suggest that grazing‐mediated MBC plays important roles in controlling *R*_s_ on the Tibetan Plateau, but more research on the mechanisms of MBC variations are needed.

Studies conducted in temperate regions have shown that GE significantly increased *R*_s_ due to corresponding increases in plant productivity, litter accumulation, and litter decomposition (Su et al. 2005; Li et al. 2013b; Gong et al. 2014). However, multiple regression analysis in the current study showed that GE‐induced variations on *R*_s_ were mainly due to changes in soil temperature, soil moisture, and MBC, but not correlated with GE‐induced changes in AGB and BGB (Table 3). It is worth noting that GE‐induced increases in AGB, BGB and litter accumulation are not necessarily accompanied by increases in litter decomposition (Lindsay and Cunningham 2009; Jing et al. 2013). Indeed, results of a recent study conducted near our study site showed that grazing increased litter decomposition by grazing‐induced higher soil temperatures (Luo et al. 2010). Those findings were possibly related to the thermal constraints on the dynamics of carbon decomposition on the cold Tibetan Plateau where the large amounts of carbon stored in the soils may be particularly susceptible to changes in microclimate and MBC (Lin et al. 2011). Our results suggest that the effects of GE‐induced changes in MBC and soil temperature and moisture on *R*_s_ may have overridden the effects of GE‐induced higher plant productivity and possibly higher litter decomposition.

### Soil respiration during the nongrowing season

We found no significant response of *R*_s_ to GE during the NGS, and this can be explained by the extremely low soil temperature and frozen soil water at our study site (Chen et al. 2015). That is, both microbial activities and root respiration are known to be affected by soil temperature and available soil moisture (Li et al. 2013a; Prem et al. 2014). This explanation is consistent with previous studies which showed that *R*_s_ during the NGS is consistently low due to the constraints on microbial and root activities (Pacaldo et al. 2013; Wang et al. 2014). However, our study did show that the amount of carbon released during the NGS could account for >20% of the annual total at both the grazed and GE blocks (Fig. 6). This finding runs counter to a previous hypothesis to the effect that *R*_s_ in winter should approach zero (Fahnestock et al. 1998), but it is consistent with some recent results showing that *R*_s_ during the NGS accounted for 14−30% of annual total *R*_s_ (Elberling 2007; Pacaldo et al. 2013). Our results suggest that carbon released during the NGS should be taken into consideration when calculating annual soil carbon flux on the Tibetan Plateau.

We further took the *R*_s_ during the NGS into consideration when calculating the annual carbon release. The GE‐induced reductions on annual soil carbon release remained significant, although GE had no significant impacts on the *R*_s_ during the NGS (Fig. 6). A previous study also showed that significantly more carbon was stored during the growing season in areas where livestock was excluded, and a more pronounced effect was seen after 3 year of GE compared with 5 year of exclusion (Chen et al. 2015). Those results−together with our new findings that GE significantly reduced soil carbon release both during the growing season and annually−indicate that GE may be an effective method for increasing carbon storage on the Tibetan Plateau.

### Grazing exclusion effects on temperature sensitivity

Grazing exclusion significantly increased Q_10_ both during the growing season and annually (Table 2), and these increases in Q_10_ imply that the carbon stored in the soils of the Tibetan Plateau may be particularly vulnerable to the climate warming (Luo and Zhou 2006). Increases in Q_10_ induced by GE also have been reported for the Yellowstone National Park, and those effects were attributed to changes in the quality of soil organic carbon (SOC) (Chuckran and Frank 2013). The quality of SOC was not measured in the current study, and this limits our ability to fully explain the GE‐induced variations in Q_10_. Nevertheless, other studies have shown that Q_10_ is closely related to soil temperature and moisture (Lin et al. 2011; Chen et al. 2015), and therefore, it is possible that the GE‐induced changes in soil microclimate also contributed to the relatively high Q_10_ values observed. Regardless of which explanation is correct, our results clearly demonstrate that the dynamics of carbon cycling in grazed and GE areas will differ if the climate changes. More specifically, GE blocks will be more susceptible to increased releases of soil carbon if the climate warming.

Grazing exclusion markedly increased Q_10_ during the start and end of the growing season (Table 2), and significantly higher values of Q_10_ were observed during those periods compared with the peak of the growing season. Our results are consistent with other studies which have shown that grazing decreased Q_10_ during the growing season (Lin et al. 2011) and that low Q_10_ values occur during the peak of the growing season (Chen et al. 2015). Previous research has shown that Q_10_ is regulated by both temperature and moisture, and higher temperatures and moisture would result in lower Q_10_ (Zhang et al. 2010) Therefore, the variations in soil temperature and moisture we observed during the growing season were likely the primary drivers for the monthly variations in Q_10_. Given these results, one would predict that if the climate warms, GE will cause the largest quantities of carbon to be released at the start and end of the growing season.

No significant GE effect on Q_10_ was found during the NGS (Table 2) when Q_10_ values were lower than those during the growing season. These results appear to be at odds with previous studies which showed that higher Q_10_ values in temperate regions occurred during the NGS (Shi et al. 2012; You et al. 2013). However, these contrasting results might be explained by the extremely low temperatures and frozen soil water during the NGS on the Tibetan Plateau (Aanderud et al. 2013; Wang et al. 2014). In fact, recent studies have shown that in cold regions soil moisture−not soil temperature−may be the primary regulator of Q_10_ during the NGS (Aanderud et al. 2013; Chang et al. 2013; Wang et al. 2014). Indeed, it is possible that the low soil temperatures and frozen soil water during the NGS on the Tibetan Plateau inhibited soil microbial activity, thus reducing Q_10_.

## Conclusions

The extremely high elevation of the alpine grassland on the Tibetan Plateau makes it unusually vulnerable to human‐induced disturbances. In this study, GE was found to significantly increase AGB and soil moisture but decrease soil temperature, MBC, and *R*_s_. The GE‐induced reductions in *R*_s_ were closely correlated with decreases in soil temperature and MBC. Compared with the grazed blocks, GE also significantly increased Q_10_, which indicates that GE blocks may have a higher potential for carbon release under future global warming scenarios. Our results highlight the importance of including interactive effects of human activities and global climate change in models of soil carbon fluxes.

## Conflict of Interest

None declared.
